# Development of a three-dimensional vitrification protocol for domestic cat cumulus-oocyte complexes and comparison with standard vitrification

**DOI:** 10.3389/fvets.2026.1807486

**Published:** 2026-04-08

**Authors:** Martina Colombo, Alessandra Mascaro, Sai Kamal Nag Bonumallu, Jasmine Fusi, Alessandro Pecile, Letizia Temerario, Antonella Mastrorocco, Sara Panseri, Gaia Cecilia Luvoni

**Affiliations:** 1 Dipartimento di Medicina Veterinaria e Scienze Animali, Università degli Studi di Milano, Lodi, Italy; 2 Dipartimento di Bioscienze, Biotecnologie e Ambiente, Università degli Studi di Bari Aldo Moro, Valenzano, Italy

**Keywords:** actin, alginate, cryopreservation, cryoprotectant, embryo, feline, maturation, viability

## Abstract

Three-dimensional (3D) systems may better mimic native tissue conditions and provide more physiologically relevant platforms for the study of cell function compared to traditional two-dimensional (2D) cultures. Cryopreservation of cells after encapsulation in 3D matrices is also likely to be a suitable alternative to traditional 2D approaches, as demonstrated with preantral ovarian follicles. Building on these findings, this study aimed to develop an alginate-based 3D vitrification protocol for domestic cat (*Felis catus*) cumulus-oocyte complexes (COCs). After determining the most suitable alginate concentration, encapsulated COCs were vitrified following a Cryotop-based protocol, with variations in exposure time to cryoprotectant (CPA) solutions. Permeation of dimethyl sulfoxide (DMSO) and ethylene glycol (EG) in COCs was quantified by gas chromatography-triple quadrupole mass spectrometry. Oocyte functional competence was assessed by *in vitro* maturation (IVM), viability, actin distribution, and embryo development rates after *in vitro* fertilization (IVF). The results showed that 1% alginate improved IVM of fresh oocytes (75% vs. 59.4% in 2D culture, *p* = 0.03), therefore was adopted for the development of the 3D vitrification protocol. Alginate-encapsulated COCs required longer exposure to CPA solutions (i.e., 2.5-fold increase compared to the standard protocol) to achieve intracellular concentrations of DMSO and EG comparable to non-encapsulated (2D) controls (*p* = 0.8). While post-IVM viability was lower in 3D vitrified oocytes (35.9%) than in standard vitrified (2D) controls (75.6%, *p* < 0.00001), among viable vitrified oocytes there were no significant differences in maturation rates (range 44.6%−65.2%, *p* = 0.14) or actin distribution (intact pattern range 76.9%−93.3%, *p* = 0.31), regardless of the vitrification protocol (2D vs. 3D). Similarly, cleavage rates following IVF did not differ between 3D vitrified and standard 2D vitrified oocytes when the longest exposure time to CPA was used (i.e., 2.5-fold increase; 10.6% vs. 27.8%, *p* = 0.08). These findings demonstrate that 3D vitrification in alginate has the potential to be employed for the cryopreservation of domestic cat COCs, and provides a proof-of-concept for further optimization. Refining 3D cryopreservation techniques in domestic cats might contribute to the optimization of translational fertility preservation strategies.

## Introduction

The advancement of three-dimensional (3D) cell culture systems has markedly improved the study of oocytes and ovarian follicles, providing a microenvironment that more closely resembles *in vivo* conditions compared with traditional two-dimensional (2D) cultures. Three-dimensional systems promote cell interactions and enhance the developmental competence of oocytes, thereby facilitating a better understanding of folliculogenesis and oocyte maturation. Combinations of compounds or single biomaterials, including fibrin, collagen, hyaluronic acid, or synthetic polymers such as polyethylene glycol, have been explored to develop tailored 3D scaffolds (1).

A growing interest in the development of bio-engineered ovaries is probably one of the main drivers behind advances in 3D systems for female reproductive medicine. Innovative bioengineering approaches, applied mostly in rodent models, have demonstrated that 3D architecture can modulate follicular physiology, allowing the reconstruction of fully functional ovaries *in vivo* (2) and the recreation of physiological events such as antrum formation and ovulation *in vitro* (3). However, only a few studies addressed the challenges of cryopreservation within 3D matrices, while this is a crucial step toward integrating the advantages of 3D culture with advanced fertility-preservation strategies.

In this context, alginate has garnered substantial attention among 3D culture matrices due to its biocompatibility, its ability to mimic the extracellular matrix (ECM), and its ease of fabrication (4). Several studies have shown that encapsulation of oocytes and ovarian follicles in alginate hydrogels not only preserves physiological cell organization but also promotes their growth and maturation (5–12). Alginate encapsulation has also been used for cryopreservation of various cell types, including animal cells.

The advantages of cryopreservation of encapsulated cells include reduced stress and manipulation, besides mechanical support and protection from cryoinjuries (13). Alginate-based 3D cryopreservation has proved successful for somatic and stem cells, ovarian follicles (14–17) and, very recently, isolated oocytes (18), encouraging further exploration of innovative cryopreservation strategies. Cryopreservation of oocytes is fundamental for fertility preservation, but it still requires optimization in many animal species, including felids, particularly for immature, germinal vesicle (GV) oocytes. Alginate-based 3D cryopreservation could represent a suitable alternative, one that has never been investigated in non-rodent animal models.

Cryopreservation of encapsulated cells presents inherent challenges, such as hydrogel size and physicochemical properties, including porosity, stiffness, and water content, which may influence cryopreserved oocyte outcomes. However, it offers practical advantages, as the hydrogel can be used directly for subsequent culture after warming. In the case of GV oocytes, in addition to the advantages of 3D cryopreservation itself, using the same matrix for subsequent 3D IVM could also allow the oocytes to benefit from the 3D culture conditions, generating a combination of positive effects that could potentially enhance biological outcomes.

Therefore, the aim of this study was to assess the effects of a newly developed 3D vitrification protocol on the structural integrity, meiotic and developmental competence of domestic cat oocytes. After optimizing alginate hydrogel for cat oocytes (Experiment I), assessing its influence on cryoprotectant (CPA) permeability (Experiment II), and applying it during vitrification, the effects of 3D vitrification on oocyte viability, nuclear maturation, cytoskeleton integrity (Experiment III) and developmental competence (Experiment IV) were assessed and compared with a standard (2D) vitrification protocol.

## Materials and methods

### Chemicals and reagents

All chemicals and reagents were purchased from the Italian branch of Merck KGaA (Darmstadt, Germany)—Merck Life Science S. r. l. (Milan, Italy), unless otherwise stated.

### Experimental design

**Figure 1:**
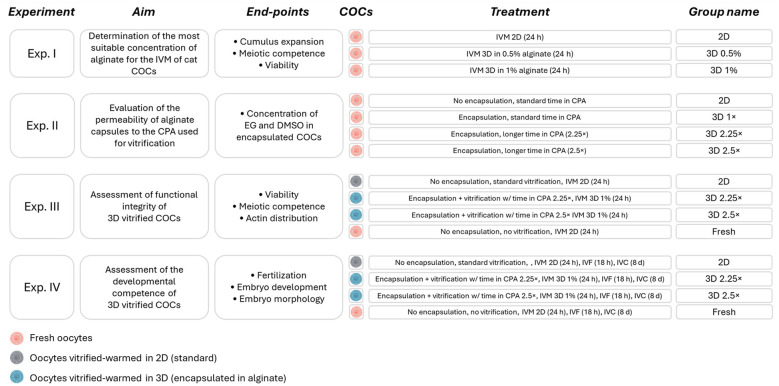
Graphical depiction of the experimental design of the study. For details, please see Paragraph 2.2. IVM, *in vitro* maturation; COCs, cumulus-oocyte complexes; 2D, two-dimensional; 3D, three-dimensional; h, hours; d, days; CPA, cryoprotectants; EG, ethylene glycol; DMSO, dimethyl sulfoxide; w/, with; 1×, standard exposure times to CPA solutions (i.e., the same as the 2D group); 2.25×, exposure times to CPA solutions increased 2.25-fold compared to the standard; 2.5×, exposure times to CPA solutions increased 2.5-fold compared to the standard; for details of exposure times to CPA please refer to Table 1.

Four experiments were performed to achieve the aim of the study (Figure 1).

According to the experiment, immature COCs were collected and used as fresh or cryopreserved by vitrification after being singly encapsulated (3D) or with a standard (2D) procedure.

In Experiment I, the effect of 3D encapsulation in different concentrations (0.5% or 1%) of alginate on oocyte viability, nuclear status, and cumulus expansion was assessed in comparison with standard 2D IVM.

Experiment II aimed to evaluate the permeability of the alginate capsules (1%, based on the results of Experiment I) to the CPA used for vitrification, i.e., dimethyl sulfoxide (DMSO) and ethylene glycol (EG). Cumulus-oocyte complexes were divided in four experimental groups: one group was exposed to CPA using the standard 2D vitrification protocol, while the other three groups consisted of encapsulated COCs undergoing exposure to CPA for different time lengths, i.e., the standard exposure time (3D 1 ×) and extended times (i.e., 2.25 or 2.5-fold increase compared to the standard time, named 3D 2.25 × and 3D 2.5×, respectively).

Experiment III assessed oocyte viability, cytoskeletal integrity and nuclear status after vitrification. Cumulus-oocyte complexes were vitrified using either the standard 2D protocol or after encapsulation in 1% alginate and prolonged exposure times to CPA (3D 2.25 × and 3D 2.5 ×). For the encapsulated groups, after warming COCs were *in vitro* matured within the same alginate capsules where they were vitrified. A group of fresh, non-cryopreserved oocytes was included as additional control. The selected endpoints were analyzed after IVM since vitrified oocytes tend to show better quality (e.g., viability) after warming and degenerate slowly during following 24 h culture (19).

Finally, Experiment IV evaluated the developmental competence of vitrified oocytes following *in vitro* fertilization (IVF). Vitrification and culture of COCs up to the IVM was performed as in Experiment III, with the same experimental groups.

### Ovaries and oocyte retrieval—Experiments I, III, IV

Ovaries from healthy queens (*Felis catus*) were harvested at random stages of the estrous cycle during routine ovariectomies or ovariohysterectomies. The study did not require ethical approval because cat ovaries were collected as byproducts from routine surgeries.

After surgery, ovaries were immediately placed in phosphate buffered saline (PBS) with a mixture of antibiotics (AB) and antimycotics (100 IU/mL of penicillin G sodium, 0.1 mg/ml of streptomycin sulfate, 0.25μg/ml of amphotericin B) and transported to the laboratory at room temperature (RT) within 2–3 h.

Ovaries were minced in PBS with 0.1% (w/v) polyvinyl alcohol (PBS-PVA) to release the oocytes (20), and only immature COCs with homogeneous darkly pigmented ooplasm completely surrounded by at least 3–4 layers of cumulus cells [Grade I (21)] were selected for the experiments (Supplementary Figure 1).

### Encapsulation in alginate matrix—Experiments I, II, III, IV

The selected COCs were encapsulated in sodium alginate (Alginic acid sodium salt from brown algae - Medium viscosity—Brookfield Viscosity: 2% in water at 25 °C = 4,470 cps—Product Number: A2033—Batch Number: SLBK5754V) following a modification of the protocol of Mastrorocco et al. (7), with manual dropping of capsules. Briefly, COCs were first washed in drops of alginate solutions [0.5%−1% alginate in IVM base medium, i.e., medium 199 supplemented with 3 mg/ml bovine serum albumin (BSA) and 0.6 mM cysteine] and subsequently dropped one-by-one, within the same alginate solution, using a manually controlled pipette set at 1 μL, into a pre-warmed (38 °C) 100 mM CaCl_2_ solution. The droplets were maintained in the CaCl_2_ solution for 5 min to allow complete capsule formation through ionic crosslinking. Capsule size was < 1 mm and was considered adequate to fit on Cryotops (Kitazato Corporation, Yanagishima, Fuji-shi, Shizuoka, Japan) for vitrification in the following experiments. Alginate was used at the concentration of 0.5% and 1% in Experiment I based on the existing literature (7, 9). Alginate was used at the concentration of 1% in Experiments II, III and IV, based on the results of Experiment I.

### Quantification of CPA permeation in encapsulated COCs—Experiment II

Bovine COCs were used as a model for the assessment of CPA permeation because of the high number of oocytes needed for each replicate (≈120 COCs/replicate). Collection and selection of COCs was performed as reported earlier (22).

Selected bovine COCs were exposed to standard equilibration [7.5% (v/v) EG and 7.5% DMSO in Medium 199, with 20% fetal bovine serum (FBS)] and vitrification solutions (15% (v/v) EG, 15% DMSO and 0.5 M sucrose in Medium 199 with 20% FBS), either using a standard protocol (2D, loading of COCs on a Cryotop) or after encapsulation in 1% alginate as described above (3D, loading of capsules containing COCs on a Cryotop). Encapsulated COCs were subjected to three CPA exposure durations: equal to the standard protocol (1×, 15 min in equilibration solution and 1.5 min in vitrification solution), 2.25 times longer (2.25 ×), or 2.5 times longer (2.5 × ; Table 1). Extended exposure times to CPA solutions were based on the results of trials assessing oocyte viability and maturation, where it was observed that shorter exposures (1.5 × and 2 ×) and further increases in exposure times (2.75 × and 3 ×) were detrimental.

**Table 1:** Exposure times to cryoprotectant solutions in different protocols [standard (1 ×) vs. increased times (2.25 × and 2.5 ×) for 3D-encapsulated cumulus-oocytes complexes].

Solution	1 ×	2.25 ×	2.5 ×
ES	3 min	6 min 45 sec	7 min 30 sec
	3 min	6 min 45 sec	7 min 30 sec
	9 min	20 min 15 sec	22 min 30 sec
VS	1 min 30 sec	3 min 22 sec	3 min 45 sec
TS	1 min	2 min 15 sec	2 min 30 sec
DS	3 min	6 min 45 sec	7 min 30 sec
WS	5 min	11 min 15 sec	12 min 30 sec

ES, equilibration solution; VS, vitrification solution; TS, thawing solution; DS, diluting solution; WS, washing solution; ES and VS are used during vitrification; TS, DS and WS are used during warming. 1×, standard exposure times to vitrification-warming solutions; 2.25×, exposure times extended 2.25-fold; 2.5×, exposure times extended 2.5-fold.

Then, concentrations of DMSO and EG were quantified in pools of ≈30 COCs (*n* = 3 pools per treatment) by gas chromatography–mass spectrometry triple quadrupole (Trace 1310; TSQ8000, Thermo Fisher Scientific) with a GC Rtx-Wax column (30 m, i.d. 0.25 mm, film thickness 0.25 μm; Restek, Bellefonte, PA, USA) and the method described in (23).

The CPA concentrations from each group were measured after washing of non-encapsulated (or mechanically de-capsulated) COCs in thawing solution (1 M sucrose in Medium 199, with 20% FBS) for 10 min to induce diffusion of CPA from the COCs to the medium. Samples were frozen at −80 °C and then analyzed. Before the analysis, they were mixed with methanol, homogenized and centrifuged at 14,100 g for 5 min; the supernatants were collected and subsequently analyzed.

Deuterated DMSO (DMSO-d6) and EG (EG-d6) were used as internal standards. The column temperature was initially held at 80 °C for 2 min, then raised at a rate of 5 °C/min to 140 °C and subsequently at a rate of 25 °C/min to 220 °C. The injector temperature was set at 240 °C in splitless mode (0.5 min). The concentration of each CPA (ppm) was obtained after normalization according to the number of COCs included in each pool (range: 25-31 COCs).

### Vitrification and warming of immature COCs—Experiments III and IV

Cumulus-oocyte complexes were vitrified by the Cryotop method (24, 25), as previously described for cat oocytes (26). Briefly, groups of 4–8 oocytes were equilibrated at RT in theequilibration solution for 15 min. Then, they were transferred into the vitrification solution, placed on Cryotop strip, removing the excess of liquid to reduce the volume as much as possible, and directly immersed into liquid nitrogen in less than 90 s. At warming, the Cryotop strip was immersed for 1 min in thawing solution at 37 °C. Vitrified COCs were retrieved and transferred for 3 min in a diluting solution containing 0.5 M sucrose in Medium 199, with 20% FBS and then for 5 min in a solution without sucrose (washing solution). Finally, they were washed again in the same solution (Medium 199 with 20% FBS) and then used for the experiments. This protocol refers to 2D-groups, where COCs are directly loaded on Cryotops.

**Figure 2:**
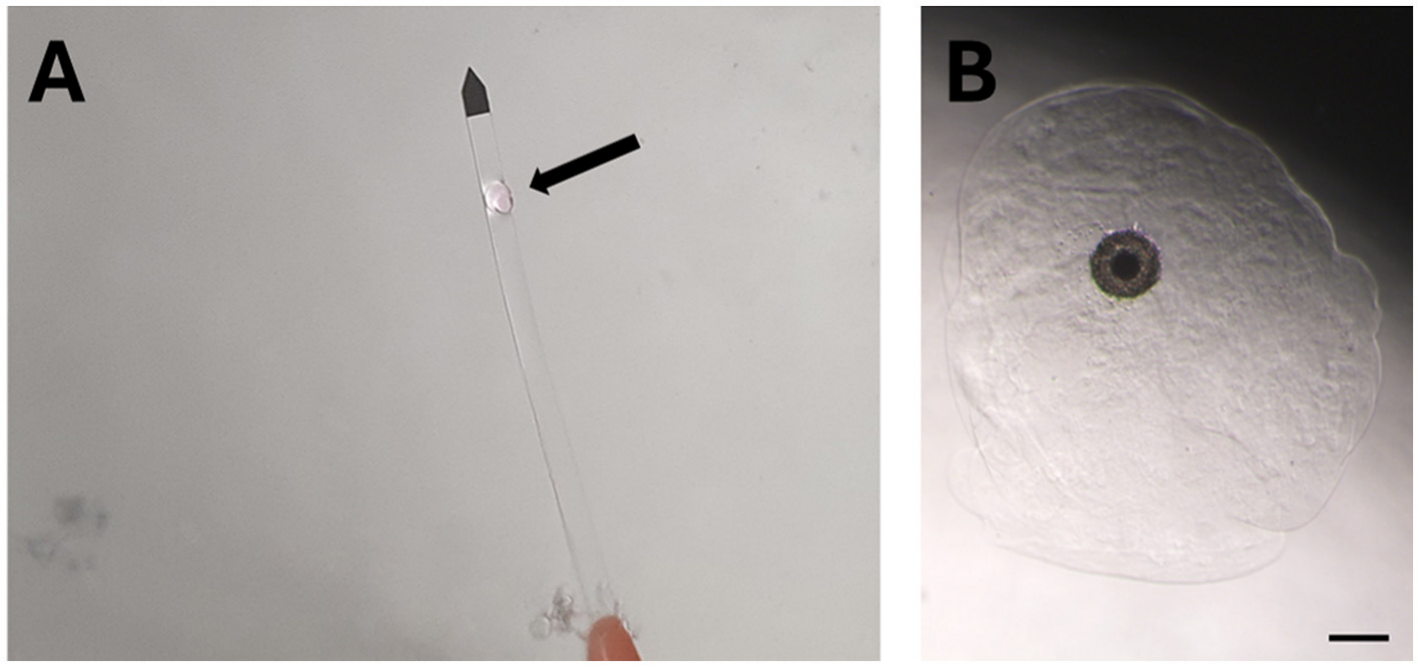
Representative pictures of three-dimensional (3D) vitrification and *in vitro* maturation (IVM) of domestic cat cumulus-oocyte complexes (COCs). **(A)** Alginate encapsulated COC (arrow) loaded on a Cryotop for 3D vitrification, after plunging into liquid nitrogen. **(B)** 3D IVM of a 3D vitrified COC, scale bar, 200 μm.

Encapsulated COCs, instead, were vitrified and warmed using the same protocol, but with extended exposure times to the equilibration, vitrification, thawing, diluting and washing solutions (2.25- or 2.5-fold increase, as depicted in Table 1). For 3D groups, alginate capsules, each containing a single COC, were moved into the different solutions and loaded onto the Cryotop (Figure 2A) with the help of an automatic pipette fitted with a pre-cut tip to facilitate the passage of the capsules.

### *In vitro* maturation—Experiments I, III, IV

Cumulus-oocyte complexes were *in vitro* matured for 24 h in groups of 3–19 in a controlled atmosphere (38.5 °C and 5% CO_2_ in air) in 400 μL microdrops of IVM medium [medium 199 supplemented with 3 mg/ml BSA, 10 ng/ml epidermal growth factor (EGF), 0.6 mM cysteine, and 0.5 IU/ml follicle-stimulating hormone+0.5 IU/ml luteinizing hormone (Pluset^®^, Calier, Spain)] (27) in 4-well dishes with the addition of distilled sterile H_2_O between the wells to maintain humidity. For the non-encapsulated groups (2D), the COCs were cultured on the bottom of the plastic wells. For the encapsulated groups (3D), COCs were *in vitro* matured in the alginate capsules (the same where they were vitrified in Experiment III and IV; Figure 2B).

In Experiment I, cumulus expansion was subjectively evaluated. Only COCs showing more than three layers of expanded cumulus cells were counted [modified from (28)]. Granulosa cells adhering to the culture plate were not considered as expansion.

At the end of the IVM, encapsulated oocytes were incubated in sodium citrate (2% in IVM base medium) for 5 min at 38.5 °C to dissolve the alginate capsules and release the COCs to be used for staining (Experiment III) or fertilization (Experiment IV).

### Assessment of viability and actin distribution—Experiments I and III

At the end of the IVM procedure, and after dissolving the alginate capsules for 3D groups, COCs were mechanically denuded using an automatic pipette and incubated in 40 μL of 5 mg/ml fluorescein diacetate (FDA) for 3 min in the dark at RT to evaluate cell viability (Experiments I and III).

Following viability assessment, in Experiment I denuded oocytes were placed on a slide, air-dried, and fixed in 80 % ethanol overnight at 4 °C for the assessment of nuclear status. In Experiment III, some oocytes were just stained for the assessment of nuclear status as in Experiment I, while others were stained both for the assessment of nuclear status and actin distribution. In the latter case, denuded oocytes were rinsed in PBS-PVA and fixed in 4% formaldehyde for 30 min. After fixation, the oocytes were washed three times in PBS-PVA for 5 min each and stored overnight at 4 °C in a parafilm-sealed 4-well plate before the evaluation of actin distribution.

ActinRed 555 ReadyProbes Reagent (Thermo Fisher Scientific—Invitrogen, Monza, Italy) was used to stain the oocytes to evaluate actin distribution, following the manufacturer's instructions, since cytoskeleton might be damaged by cryoinjuries (29), as well as 2D culture (30). Fixed oocytes were permeabilized with 0.5% Triton X-100 for 15 min and rinsed in PBS-PVA for 5 min. After that, they were incubated with the dye (1:8) for 30 min in the dark at RT, followed by a 5-min wash in PBS-PVA. After the wash, the oocytes were placed onto microscope slides and stained with Hoechst as described below (Paragraph 2.11).

Finally, the stained oocytes were examined under an epifluorescence microscope (Eclipse E600, Nikon) equipped with a Nikon DS-F12 digital camera at 400 × magnification.

**Figure 3:**
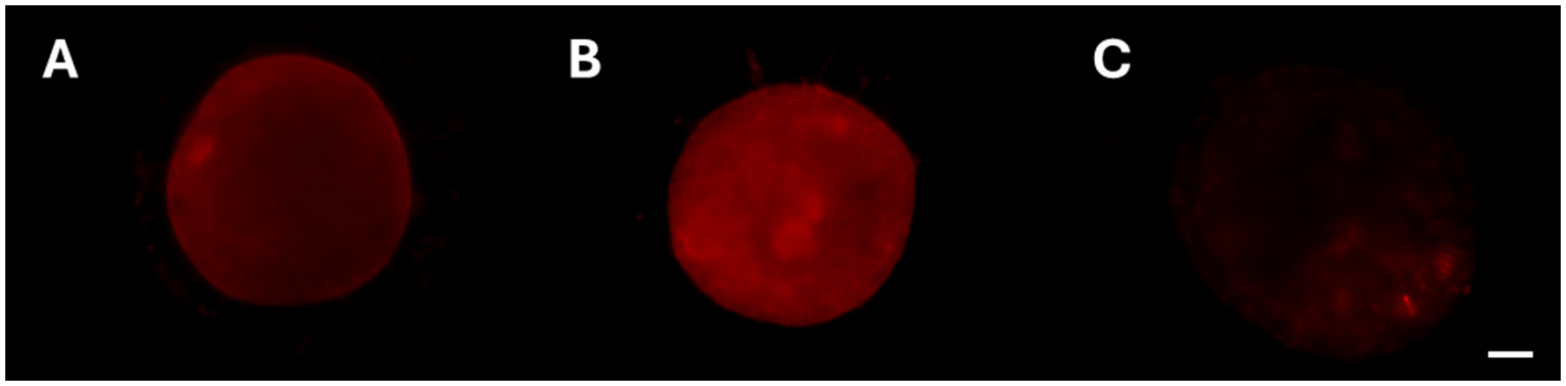
Representative fluorescence micrographs of actin distribution patterns observed in this study in domestic cat oocytes. **(A)** Pattern 1, diffuse with slightly greater concentration beneath the oolemma; **(B)** Pattern 2, uniformly distributed; **(C)** Pattern 3, abnormally distributed (mottled, discontinuous, fading). Scale bar: 20 μm.

For cytoskeletal assessment, actin distribution patterns were classified into four distinct categories, adapted from the classifications described by (29) and (31), as follows (Figure 3):

- Pattern 1: diffuse with slightly greater concentration beneath the oolemma;
- Pattern 2: uniformly distributed;
- Pattern 3: abnormally distributed (mottled, discontinuous, fading);
- Pattern 4: apparent indentation.

### Epididymal sperm recovery, freezing-thawing and *in vitro* fertilization—Experiment IV

*In vitro* fertilization of COCs was performed with frozen feline epididymal spermatozoa obtained after routine orchiectomy of adult tomcats (*n* = 2). All the IVF replicates were performed using the same batch of spermatozoa (1 straw/replicate).

Testes were collected after routine surgery. The epididymides were dissected from isolated testicles and placed in a Petri dish in HEPES-buffered Medium 199. Spermatozoa were obtained from vas deferens and cauda epididymis by slicing with a blade at RT. Spermatozoa from the two cats were pooled, and subjective motility and concentration by Bürker chamber were determined.

Spermatozoa were frozen at the final concentration of 25,000 motile spermatozoa/ μL according to the Uppsala method originally developed for canine semen (32), using egg yolk-Tris-based extenders containing Equex, which was also tested in cat epididymal spermatozoa (33, 34). Briefly, collected spermatozoa were centrifuged (700 g, 5 min), the supernatant was removed and spermatozoa were diluted at RT with the first freezing extender. After cooling at 4 °C for 3 h, the second freezing extender was added [overall composition of freezing extender: Tris buffer with final concentrations of 5% glycerol, 1% Equex STM (Nova Chemical Sales Inc., Scituate, MA, USA) and 20% egg yolk (32)], and spermatozoa suspension was loaded into 0.2-ml straws, which were frozen in liquid nitrogen vapors before plunging into the liquid and storage.

For thawing, straws were exposed to air for 10 s and then immersed in a water bath at 37 °C for 30 s.

For fertilization, COCs were washed twice and transferred into 250 μL microdrops of fresh IVF medium [Medium 199 supplemented with 3 mg/ml BSA, 0.1 mg/ml cysteine, 0.25 mg/ml sodium pyruvate, 0.6 mg/ml sodium lactate, 0.15 mg/ml L-glutamine, 0.055 mg/ml gentamicin and 2.2 IU/ml heparin (27)] covered by 300 μL mineral oil in 4-well dishes. Thawed spermatozoa, previously centrifuged (700 g, 5 min) and resuspended in IVF medium, were added to the microdrops containing the oocytes to reach 400 μL volume with a final concentration of 1 x 10^6^ motile spermatozoa/mL. Oocytes and spermatozoa were co-incubated in a controlled atmosphere (38.5 °C and 5% CO_2_ in air) for 18 h.

### *In vitro* embryo culture—Experiment IV

After IVF, all oocytes were gently washed in *in vitro* culture (IVC) medium (Ham's F-10 supplemented with 5% FBS, 0.11 mg/ml sodium pyruvate, 0.075 mg/ml L-glutamine, 0.6 mg/ml gentamicin) to remove spermatozoa and residual cumulus cells with the help of a stripper micropipette (The Stripper, BioTipp, Waterford, Ireland) equipped with a 125 μm tip (RI-Tip, Gynemed, Lensahn, Germany). Presumptive embryos were moved to 500 μL IVC medium covered by 300 μL mineral oil in 4-well dishes, where they were cultured in group for up to 8 days in a controlled atmosphere (38.5 °C, 5% CO_2_). The medium was not changed during embryo culture. During IVC, assessment of embryo development was performed on day 2, 5, 7, and 8 post-fertilization under an inverted microscope at 100 × magnification (Axiovert 100, Zeiss).

Two days after IVF, uncleaved oocytes were deprived of remaining cumulus cells and bound spermatozoa by mechanical displacement with a stripper micropipette equipped with a 125 μm tip, placed on a slide, air-dried, and fixed in 80% ethanol overnight at 4 °C. Growing embryos were cultured until day 8 or until they showed signs of degeneration and then fixed on a slide as above.

Embryo quality was subjectively evaluated according to the International Embryo Transfer Society (IETS) Manual (35), based on morphological integrity of embryos (Code 1—excellent or good; Code 2—fair; Code 3—poor; Code 4—dead or degenerating), as previously applied in cats (20).

### Assessment of maturation, fertilization and embryonic developmental rates—Experiments I, III, IV

Chromatin configuration was evaluated by a bis-benzimide (Hoechst 33,342) staining to ascertain the nuclear status of the oocytes (Experiments I and III), or the fertilization pattern or embryo developmental stage of embryos based on the number of blastomere nuclei (Experiment IV). Briefly, oocytes or embryos placed on a slide were covered by 10 μl of Hoechst working solution (0.01 mg/mL). After 5 min of incubation in the dark, the Hoechst solution was removed and the slides were covered with antifading solution and a coverslip. Finally, stained oocytes (Experiment I) and embryos (Experiment IV) were observed under a fluorescence microscope (Axiovert 100, Zeiss) at 400 × magnification.

Oocyte chromatin configurations were classified as follows (36, 37):

- Germinal vesicle (GV): identification of nucleolus and very fine filaments of chromatin;
- Germinal vesicle breakdown–anaphase I (GVBD-AI): identification of different patterns of chromatin condensation (GVBD) or identification of bivalents (AI);
- Telophase I–metaphase II (TI-MII): identification of two groups of chromosomes moving to opposite ends of meiotic spindle (TI) or two sets of chromosomes clearly visible (MII);
- Degenerated: collapsed nucleus or irregular nuclear conformation.

For Experiment IV, the total number of fertilized oocytes was calculated as the sum of fertilized oocytes (i.e., uncleaved but showing pronuclei) and cleaved embryos. For the assessment of embryonic development, cleaved embryos (2–4 cells), morulae, and blastocysts were recorded (Supplementary Figure 2). An embryo was considered a morula when it had ≥16 cells without a blastocoel, or a blastocyst when it had ≥50 cells with a blastocoel (38).

### Statistical analysis

In Experiment I, III and IV, data were analyzed by Fisher's exact test. In Experiment II, CPA concentrations were analyzed by one-way ANOVA (data were normally distributed according to Shapiro-Wilk test and homoscedastic according to Levene's test), followed by Tukey's *post-hoc* test. The level of significance was set at *p* ≤ 0.05.

## Results

### Experiment I

The cumulus expansion, nuclear status and viability of fresh cat oocytes after IVM in 3D capsules produced with different concentrations of alginate are reported in Table 2. Three-dimensional IVM significantly improved cumulus expansion rates (*p* < 0.00001 vs. 2D), with no differences between the two tested alginate concentrations (*p* = 0.09). Partial meiotic resumption and degeneration rates did not differ among the tested conditions, but culture in 3D alginate 1% capsules significantly improved full maturation rates compared to control, 2D IVM (*p* = 0.03). No differences were found in viability rates (*p* = 0.29). Based on full maturation rates, 1% alginate was chosen for the development of the 3D vitrification protocol.

**Table 2:** Cumulus expansion, nuclear status and viability of fresh domestic cat oocytes following *in vitro* maturation (IVM) in standard (two-dimensional, 2D) or three-dimensional (3D) culture (10 replicates).

Treatment	*COCs n*.	Cumulus expansion *n*. (%)	Immaturity *n*. (%)	Partial meiosis resumption *n*. (%)	Full maturation *n*. (%)	Deg/N.A. *n*. (%)	Viability *n*. (%)
2D	101	2 (2)^a^	1 (1)^a^	21 (20.8)^a^	60 (59.4)^a^	19 (18.8)^a^	89 (88.1)^a^
3D 0.5%	112	35 (31.3)^b^	5 (4.5)^a^	15 (13.4)^a^	72 (64.3)^a, b^	20 (17.9)^a^	97 (86.6)^a^
3D 1%	104	21 (20.2)^b^	1 (1)^a^	12 (11.5)^a^	78 (75)^b^	13 (12.5)^a^	95 (91.3)^a^

Different superscripts (a, b) within the same column indicate significant differences among groups (*p* < 0.05).

COCs, cumulus-oocyte complexes; 2D, standard IVM; 3D, IVM in microcapsules of alginate at the concentration of 0.5% (3D 0.5%) or 1% (3D 1%); Immaturity, germinal vesicle (GV) oocytes; Partial meiosis resumption, oocytes in germinal vesicle breakdown (GVBD), metaphase I (MI), anaphase I (AI); Full maturation, oocytes in telophase I (TI), metaphase II (MII); Deg/N.A, degenerated oocytes/not assessable, parthenogenetically activated oocytes were considered as degenerated.

### Experiment II

To assess the effect of alginate encapsulation on the permeation of CPA in COCs, concentrations of EG and DMSO were quantified in bovine COCs (Table 3). Concentrations of CPA in encapsulated COCs (3D 1×, 3D 2.25 × and 3D 2.5 ×) increased when the exposure time to CPA solutions was extended. Concentration of EG in 3D 2.5 × was significantly higher than in 3D 1 × (*p* = 0.007), but similar to 3D 2.25 × (*p* = 0.08) and to 2D, non-encapsulated COCs (*p* = 1). Concentration of EG in 3D 1 × was significantly lower than in 2D (*p* = 0.008). Similar results were obtained with DMSO. Although there were no differences in the concentration of DMSO between 3D 1 × and 3D 2.25 × (*p* = 0.38), only the 3D 2.25 × and 3D 2.5 × showed similar concentrations to standard 2D COCs (2D, *p* = 0.84). The highest values were observed in 3D 2.5×, being significantly higher than 3D 1 × (*p* = 0.005). These results suggested that alginate encapsulation slowed down CPA permeation in encapsulated COCs and that a 2.5-fold increase in exposure time to EG and DMSO solutions was effective in achieving the same level of cryoprotection in the 3D system as in the standard 2D.

**Table 3:** Concentration of ethylene glycol (EG) and dimethyl sulfoxide (DMSO) in bovine cumulus-oocyte complexes (COCs) after exposure to cryoprotectant solutions in different conditions.

Treatment	EG (ppm)	DMSO (ppm)
2D	168.51 ± 21.72 ^a^	150.01 ± 23.10 ^a, b^
3D 1 ×	81.24 ± 10.59 ^b^	80.53 ± 8.35 ^c^
3D 2.25 ×	114.68 ± 21.04 ^a, b^	109.56 ± 19.18 ^a, c^
3D 2.5 ×	169.64 ± 34.00 ^a^	164.04 ± 27.79 ^b^

Data are presented as mean ± SD of 3 different pools (normalized for 30 COCs/pool). Different superscripts (a, b, c) within the same column indicate significant differences among groups (*p* ≤ 0.05).

ppm, parts per million; 2D, COCs exposed to cryoprotectant solutions in standard conditions (without alginate encapsulation, control); 3D, COCs exposed to cryoprotectant solutions for different time lengths after encapsulation in sodium alginate; 1×, standard exposure times to cryoprotectant solutions (i.e., the same as the 2D group); 2.25×, exposure times to cryoprotectant solutions increased 2.25-fold compared to the standard; 2.5×, exposure times to cryoprotectant solutions increased 2.5-fold compared to the standard.

### Experiment III

The viability and nuclear status of vitrified cat oocytes after IVM are reported in Table 4. As it is known, vitrification affects oocytes viability, and all the groups of vitrified oocytes showed lower values compared to fresh oocytes (*p* = 0.03). Standard vitrification resulted in higher viability than 3D vitrification (*p* < 0.00001), while no differences were observed between 3D 2.25 × and 3D 2.5 × (*p* = 1). Regarding the nuclear status, feline COC resumed meiosis at a similar rate after 3D vitrification, regardless of the exposure time to CPA solutions (3D 2.25 × and 3D 2.5×, *p* = 0.61), but 3D 2.25 × had lower rates than standard vitrified and fresh COCs (*p* = 0.03). Full maturation was achieved by all the groups, without significant differences between 3D 2.25 × and 3D 2.5 × (*p* = 0.51). The longest exposure time to CPA (3D 2.5 ×) showed no differences in maturation and degeneration rates compared to the control (2D, *p* = 0.2), but the shortest (3D 2.25 ×), gave significantly higher degeneration and lower maturation rates (*p* = 0.03 vs. 2D). Moreover, considering only viable oocytes, there were no differences among all the groups concerning full maturation (*p* = 0.14), suggesting that surviving vitrified oocytes retained their meiotic competence regardless of the cryopreservation procedure.

**Table 4:** Viability and nuclear status of vitrified [standard protocol or three-dimensional (3D) vitrification] and fresh domestic cat oocytes after *in vitro* maturation (at least 6 replicates per group).

Treatment	COCs *n*.	Viability *n*. (%)	Immaturity *n*. (%)	Partial meiosis resumption *n*. (%)	Full maturation *n*. (%)	Deg/N.A. *n*. (%)	Mature/viable *n*. (%)
2D	86	65 (75.6)^a^	6 (7)^a, b^	23 (26.7)^a, b^	29 (33.7)^a, b^	28 (32.6)^a^	29/65 (44.6)^a^
3D 2.25 ×	64	23 (35.9)^b^	7 (10.9)^a, b^	8 (12.5)^c^	11 (17.2)^c, d^	38 (59.4)^b, c^	11/23 (47.8)^a^
3D 2.5 ×	64	23 (35.9)^b^	8 (12.5)^a^	10 (15.6)^b, c^	15 (23.4)^a, d^	31 (48.4)^a, c^	15/23 (65.2)^a^
Fresh	52	47 (90.4)^c^	1 (1.9)^b^	18 (34.6)^a^	26 (50)^b^	7 (13.5)^d^	26/47 (55.3)^a^

Different superscripts (a, b, c, d) within the same column indicate significant differences among groups (*p* < 0.05).

COCs, cumulus-oocyte complexes; Immaturity, germinal vesicle (GV) oocytes; Partial meiosis resumption, oocytes in germinal vesicle breakdown (GVBD), metaphase I (MI), anaphase I (AI); Full maturation, oocytes in telophase I (TI), metaphase II (MII); Deg/N.A., degenerated oocytes/not assessable; parthenogenetically activated oocytes were considered as degenerated; 2D, COCs vitrified in standard conditions (without alginate encapsulation, control); 3D 2.25×, COCs vitrified after alginate encapsulation, with exposure times to cryoprotectants extended 2.25-fold compared to the standard; 3D 2.5×, COCs vitrified after alginate encapsulation, with exposure times to cryoprotectants extended 2.5-fold compared to the standard; Fresh, control, non-cryopreserved cumulus-oocyte complexes.

Concordant results were observed for actin distribution (Table 5). Intact actin distribution (pattern 1) was predictably the highest in fresh oocytes. Pattern 1 was also significantly higher in standard 2D vitrified oocytes than in 3D ones (*p* = 0.0002), with no differences between the two exposure times (3D 2.25 × and 3D 2.5×, *p* = 0.47). Pattern 3, which was more commonly found in degenerated oocytes, was significantly higher in 3D 2.25 × and 3D 2.5 × compared to the controls (*p* < 0.00001). Again, considering only viable oocytes, there were no noticeable differences between standard and 3D vitrification (*p* = 0.38) and no differences between 3D 2.5 × and fresh oocytes (*p* = 0.43), suggesting that surviving oocytes maintained proper actin distribution, especially after the longest exposure time to CPA in the 3D system. Only few oocytes showed pattern 2 upon microscopical observation, and none showed pattern 4, with no statistically significant differences.

**Table 5:** Actin distribution in vitrified [standard protocol or three-dimensional (3D) vitrification] and fresh domestic cat oocytes after *in vitro* maturation (at least 6 replicates per group).

Treatment	COCs *n*.	Pattern 1 *n*. (%)	Pattern 2 *n*. (%)	Pattern 3 *n*. (%)	Pattern 4 *n*. (%)	Pattern 1/viable *n*. (%)
2D	51	37 (72.5)^a^	3 (5.9)^a^	11 (21.6)^a^	0 (0)^a^	37/42 (88.1)^a, b^
3D 2.25 ×	43	10 (23.3)^b^	2 (4.7)^a^	31 (72.1)^b^	0 (0)^a^	10/13 (76.9)^b^
3D 2.5 ×	43	14 (32.6)^b^	1 (2.3)^a^	28 (65.1)^b^	0 (0)^a^	14/15 (93.3)^a, b^
Fresh	52	46 (88.5)^c^	0 (0)^a^	6 (11.5)^a^	0 (0)^a^	46/47 (97.9)^a^

Different superscripts (a, b, c) within the same column indicate significant differences among groups (*p* < 0.05).

COCs, cumulus-oocyte complexes; Actin Pattern 1, diffuse with slightly greater concentration beneath the oolemma; Actin Pattern 2, uniformly distributed; Actin Pattern 3, abnormally distributed (mottled, discontinuous, fading); Actin Pattern 4, apparent indentation; 2D, COCs vitrified in standard conditions (without alginate encapsulation, control); 3D 2.25×, COCs vitrified after alginate encapsulation, with exposure times to cryoprotectants extended 2.25-fold compared to the standard; 3D 2.5×, COCs vitrified after alginate encapsulation, with exposure times to cryoprotectants extended 2.5-fold compared to the standard; Fresh: control, non-cryopreserved cumulus-oocyte complexes.

### Experiment IV

Fertilization and embryonic developmental rates of standard and 3D vitrified COCs, as well as those of fresh controls, are shown in Table 6. All the groups underwent successful fertilization and cleavage, although with some differences. As expected, vitrification affected the developmental competence of vitrified oocytes, especially at late *in vitro* stages of development. Indeed, while fertilization and cleavage of 2D vitrified oocytes were statistically comparable to those of fresh COCs but showed a tendency to be higher in fresh COCs (*p* = 0.056), few morulae and no blastocysts were obtained (*p* = 0.001, 2D vs. fresh COCs). Degeneration rates also followed the same trend and were significantly higher in standard 2D vitrified oocytes than in fresh COCs (*p* = 0.03).

**Table 6:** Fertilization, embryonic development and degeneration rates of vitrified [standard protocol or three-dimensional (3D) vitrification] and fresh domestic cat oocytes (6 replicates).

Treatment	COCs	Fertilization^†^	Embryo development			Deg./N.A.
			**Cleavage (2–4 cells)**	**Morula**	**Blastocyst**	
	***n***.	***n***. **(%)**	***n***. **(%)**	***n***. **(%)**	***n***. **(%)**	***n***. **(%)**
2D	36	11 (30.6)^a^	10 (27.8)^a, c^	2 (5.6)^a^	0 (0)^a^	17 (47.2)^a^
3D 2.25 ×	40	3 (7.5)^b^	2 (5)^b^	1 (2.5)^a^	0 (0)^a^	33 (82.5)^b^
3D 2.5 ×	47	5 (10.6)^b^	5 (10.6)^b, c^	0 (0)^a^	0 (0)^a^	38 (80.9)^b^
Fresh	58	30 (51.7)^a^	29 (50)^a^	24 (41.4)^b^	13 (22.4)^b^	15 (25.9)^c^

Different superscripts (a, b, c) within the same column indicate significant differences among groups (*p* < 0.05).

COCs, cumulus-oocyte complexes; 2D, COCs vitrified in standard conditions (without alginate encapsulation, control); 3D 2.25×, COCs vitrified after alginate encapsulation, with exposure times to cryoprotectants extended 2.25-fold compared to the standard; 3D 2.5×, COCs vitrified after alginate encapsulation, with exposure times to cryoprotectants extended 2.5-fold compared to the standard; Fresh, control, non-cryopreserved cumulus-oocyte complexes; Deg/N.A., degenerated oocytes/not assessable.

^†^The fertilization rate was calculated as the sum of fertilized oocytes (i.e., uncleaved but showing pronuclei) and cleaved embryos.

Embryonic development of 3D vitrified oocytes was further affected, with fertilization and cleavage rates being significantly lower than for standard 2D vitrification, except for the cleavage rate of 3D 2.5×, which was similar to the 2D vitrification group (*p* = 0.08). No blastocysts were observed in the 3D vitrified groups, which also showed the highest degeneration rate (*p* = 0.001 vs. 2D and *p* < 0.00001 vs. fresh COCs).

The evaluation of embryo morphological quality (Table 7) also gave better results for fresh COCs, as expected. The latter showed higher proportions of code 1 (excellent or good) embryos than all the vitrified groups (*p* = 0.002 on day 2 and *p* = 0.04 on day 5; no statistical significance on days 7 and 8, likely due to the small number of embryos). No differences were observed between 3D 2.25 × and 3D 2.5 × at any time-point, nor between 3D and 2D except that 2D vitrified oocytes tended to show higher morphological quality on day 2 (*p* = 0.048).

**Table 7:** Morphological quality classification of embryos obtained *in vitro* from vitrified [standard protocol or three-dimensional (3D) vitrification] and fresh domestic cat oocytes (6 replicates).

Day of embryo culture	Treatment	COCs *n*.	Code 1 (excellent or good)	Code 2 (fair)	Code 3 (poor)	Code 4 (uncleaved, dead or degenerating)
			***n***. **(%)**	***n*** **. (%)**	***n*** **. (%)**	***n*** **. (%)**
Day 2	2D	36	6 (16.7)^a^	4 (11.1)^a, b^	4 (11.1)^a^	22 (61.1)^a^
	3D 2.25 ×	40	1 (2.5)^b^	1 (2.5)^a, b^	1 (2.5)^a, b, c^	37 (92.5)^b^
	3D 2.5 ×	47	0 (0)^b^	6 (12.8)^a^	0 (0)^b, c^	41 (87.2)^b^
	Fresh	58	28 (48.3)^c^	1 (1.7)^b^	0 (0)^c^	29 (50)^a^
Day 5	2D	12	2 (16.7)^a^	6 (50)^a, b^	3 (25)^a^	1 (8.3)^a, b^
	3D 2.25 ×	3	0 (0)^a^	0 (0)^a^	1 (33.3)^a, b^	2 (66.7)^a^
	3D 2.5 ×	6	0 (0)^a^	1 (16.7)^a, b^	4 (66.7)^a^	1 (16.7)^a, b^
	Fresh	29	20 (69)^b^	9 (31)^b^	0 (0)^b^	0 (0)^b^
Day 7	2D	6	1 (16.7)^a^	3 (50)^a^	1 (16.7)^a^	1 (16.7)^a^
	3D 2.25 ×	1	0 (0)^a^	0 (0)^a^	1 (100)^a^	0 (0)^a^
	3D 2.5 ×	1	0 (0)^a^	0 (0)^a^	1 (100)^a^	0 (0)^a^
	Fresh	29	11 (37.9)^a^	14 (48.3)^a^	4 (13.8)^a^	0 (0)^a^
Day 8	2D	5	0 (0)^a^	1 (20)^a^	3 (60)^a^	1 (20)^a^
	3D 2.25 ×	0	0 (0)^a^	0 (0)^a^	0 (0)^a^	0 (0)^a^
	3D 2.5 ×	0	0 (0)^a^	0 (0)^a^	0 (0)^a^	0 (0)^a^
	Fresh	29	13 (44.8)^a^	8 (27.6)^a^	5 (17.2)^a^	3 (10.3)^a^

Codes for embryo grading were given following the Manual of the International Embryo Transfer Society (IETS). Different superscripts (a, b, c) within the same column indicate significant differences among groups (*p* < 0.05).

COCs, cumulus-oocyte complexes; 2D, COCs vitrified in standard conditions (without alginate encapsulation, control); 3D 2.25×, COCs vitrified after alginate encapsulation, with exposure times to cryoprotectants extended 2.25-fold compared to the standard; 3D 2.5×, COCs vitrified after alginate encapsulation, with exposure times to cryoprotectants extended 2.5-fold compared to the standard; Fresh, control, non-cryopreserved cumulus-oocyte complexes; Deg/N.A, degenerated oocytes/not assessable.

## Discussion

The use of 3D scaffolds for cryopreservation, particularly alginate-based hydrogels, is gaining popularity for a wide range of cell types to protect them from cryoinjuries and manipulation stress (15). The hydrogel network can bind to water molecules, limiting their mobility and lowering the amount of free water, consequently reducing the risk of ice crystal formation. Moreover, the hydrogel can slow down the exchange of CPA with the surrounding solution, thereby minimizing osmotic stress on the cells (39, 40). The use of vitrification as ice crystal-free cryopreservation techniques for encapsulated cells might particularly benefit from these alginate properties, but the development of such a technique presents inherent challenges.

Vitrification usually requires high viscosity of the sample, correlated with CPA concentration and behavior, as well as small volumes, to improve heat transfer and facilitate higher cooling rates. It also requires rapid cooling, often achieved by direct immersion into liquid nitrogen by a direct immersion into liquid nitrogen (41). Besides avoiding ice-related injuries, vitrification offers other advantages, including ease and speed of execution and better suitability for lipid-rich cells (42), such as feline oocytes. However, when vitrification of macroscopic structures is performed, all these factors should be considered. While the volume of isolated oocytes is limited, alginate capsules are larger, which decreases cooling and warming rates. Moreover, the hydrogel is permeable (43), but the speed of intracellular permeation of CPA changes compared to isolated cells and depends on the hydrogel features (e.g., total dimension, pore size). These represent specific challenges that need to be addressed in the development of a tailored 3D cryopreservation protocol. However, considering the success of 3D vitrification on ovarian follicles in different species (14–17) and more recently on murine oocytes (18), the hypothesis of the present study was that 3D vitrification could also be developed for cat COCs.

In this study, the hydrogel composition was chosen based on the outcomes of oocyte IVM. Since 3D cryopreserved cells can be cultured in the same 3D matrix after warming, it is crucial that the matrix is beneficial for incubated cells. Our previous studies already demonstrated alginate suitability for cat oocytes (5, 6, 9), but they employed group culture (i.e., encapsulation of more oocytes in the same alginate capsule) as a strategy to exploit the beneficial exchange of paracrine factors, therefore larger capsules were used. To increase the chances of vitrification and simplify manipulation, smaller hydrogels were used in this study. Moreover, based on the positive results obtained in other studies (7, 8), single COC encapsulation was applied to better resemble the follicular niche. Since matrix stiffness can influence cellular outcomes (44), different alginate concentrations were tested. When produced with 1% alginate, the 3D system improved fresh oocyte cumulus expansion and IVM rates. For this reason, it was considered suitable for maintaining feline oocytes and selected for the development of 3D vitrification.

Besides stiffness, alginate composition can influence matrix permeability (45). Variations in alginate microstructure, such as the ratio of mannuronic (M) to guluronic (G) blocks, crosslinking density, and overall polymer concentration, affect pore size and the diffusion properties of the hydrogel. These parameters determine how easily small molecules, including CPA, cross the matrix and permeate the encapsulated COCs. The increased exposure time to CPA applied in the present study was necessary to allow their intracellular permeation and obtain, at least to some extent, oocyte survival, maturation and development. Although short exposure time to CPA is reported for encapsulated bovine follicles (46), inadequate permeation could affect cell survival and ice formation. The exposure time to CPA influences the ratio of free water available in the hydrogel, as well as water replacement with CPA in encapsulated cells (18, 47). If CPA permeation is inadequate, there is no sufficient reduction of freezable water within the alginate matrix itself, contributing to an unfavorable vitrification environment. Moreover, cells may undergo incomplete dehydration, leaving a proportion of free water that can crystallize during cooling or warming, thereby increasing cryoinjury risk.

To indirectly assess scaffold permeability, the permeation of EG and DMSO in COCs was quantified by gas chromatography-triple quadrupole mass spectrometry. Although this method has been reported for residual CPA quantification (23), its application to estimate CPA presence inside cells is novel, particularly in COCs. A limit of the method employed in this study is the use of bovine COCs for this analysis, a choice that was made due to the sample size needed to perform it, which was not achievable with feline samples. Although the absolute values of intracellular concentrations of EG and DMSO might differ in cat samples, since the quantification of the standard 2D protocol was also performed on bovine COCs, the proportions of permeation (i.e., lower concentrations in 3D 1 × and higher and similar to 2D in 3D 2.5 ×) are likely representative. The biological outcomes observed in feline oocytes in Experiment III and IV seem to confirm the validity of these observations, since more similarities were found between 3D 2.5 × and the 2D standard vitrification protocol.

Vitrification may damage oocytes, inducing apoptotic death and hindering their ability to properly develop into embryos (48). In the present study, a decrease in oocyte viability after warming and IVM was evident in all the cryopreserved groups and more marked in 3D vitrified oocytes. Viability of encapsulated ovarian follicles decreased after vitrification in the bovine model (46), whereas it was maintained or even improved in mouse follicles and oocytes (14, 16, 18). Besides the employed protocol, these differences could be due to the species-specific characteristics of the studied gametes. Compared to murine gametes, bovine and feline gametes are rich in cytoplasmic lipid droplets, which render them more cryosensitive (49), and may make the development of 3D cryopreservation protocols more challenging in domestic mammals. When actin distribution was assessed, the results resembled those of viability. Although 3D systems should benefit cytoskeleton structure by preventing cell flattening on culture supports (30), it can be hypothesized that this was not enough for the cells to recover from cryopreservation-induced alterations. Indeed, temperature decrease particularly affects the microtubules, but it may also damage adjacent microfilaments, whose stability may be further compromised by CPA exposure (50). The effects on actin hereby observed could be specifically attributed to microfilament alterations, with possible consequences on spindle rotation, polar body extrusion, pronuclear migration and cytokinesis (50).

Despite these findings, all the vitrified groups achieved full maturation and embryo development *in vitro*, and those with the longest exposure time to CPA (3D 2.5 ×) showed similar maturation and cleavage rates to standard, 2D vitrified COCs. When analyzing the features of surviving oocytes in Experiment III, 3D vitrification with the longest CPA exposure tended to better preserve oocyte morphofunctional integrity. Vitrified oocytes 3D 2.5 × showed the highest numerical values of full maturation and pattern 1 actin among the cryopreserved groups, suggesting that 3D vitrification and 3D IVM may help maintain oocyte ultrastructure and meiotic competence, counteracting the aforementioned damages induced by cryopreservation and 2D culture (30, 50). However, this was not directly reflected in embryo developmental rates. Oocytes that successfully resume meiosis and display preserved actin organization may still harbor subtle intracellular alterations that become evident only at later stages, as cytoplasmic maturation events beyond nuclear progression are required for embryo development (51, 52). Although structurally better-preserved oocytes may be expected to have better functionality, these two aspects do not always correlate, and this disconnect was evident in the present study.

Although 3D 2.5 × COCs cleaved at similar rates to control (2D) vitrified COCs, their morphological quality was suboptimal, and no development to more advanced stages was achieved, as in the other vitrified groups, confirming the inherent challenges of embryo production from cryopreserved feline oocytes (48, 53). These results differ from those reported by Shen et al. in the only currently available study on vitrification of encapsulated oocytes (18). Using mature murine oocytes, their study reported significant improvements in embryo development after vitrification in alginate capsules with low CPA concentrations (18). Besides the species-specific differences, with murine oocytes being generally more cryotolerant than feline ones, the nuclear stage of the oocytes may also have played a role (54, 55). Improving feline vitrified oocyte embryo development remains an ambitious goal, and the lack of *in vitro* development until the blastocyst stage probably remains the major obstacle to overcome (53). The present findings suggest that while 3D vitrification may support structural preservation, particularly in actin organization, oocyte functionality remained impaired. Although resumption of meiosis was relatively conserved, further refinements are necessary for these structural benefits to translate into improved developmental competence.

Overall, 3D systems, particularly those employing alginate-based matrices, could represent a significant advance in the preservation of female fertility. The present study focused on CPA exposure time, but further analyses are warranted. Studies exploring CPA loading methods, such as the use of microfluidic systems (56), could offer new perspectives for improving CPA permeation and cryoprotection of encapsulated cells. Although in the present work warming times were proportional to those used for vitrification, the transition from subzero values to physiological ones is another critical step that deserves in-depth investigation. Avoiding ice recrystallization during warming is essential to guarantee cell survival and function. For instance, the use of nanowarming was effective for encapsulated mouse follicles after vitrification, leading to the birth of healthy pups (16). In-depth characterization of hydrogels, including stiffness and elastic properties, as well as porosity and ultrastructure, is also needed to understand and model their correlation with biological outcomes. Finally, fine-tuning of the scaffold or the use of composite matrices [e.g., alginate derivatives, where several macromolecules can be added (45), such as some typical of the ECM] could also offer opportunities for improvement. The development of this research line in model animals, such as the domestic cat, might support applications in other species, including humans and wild felids (57–59). Continued optimization of scaffold composition and cryopreservation-warming protocols holds promise for improving cryopreserved oocyte quality and enhancing the outcomes of assisted reproductive technologies for biodiversity preservation.

## Conclusions

Cryopreservation of alginate-encapsulated domestic cat COCs deserves further investigations. Alginate is a suitable matrix for IVM, but specific modifications to the standard protocols are required for its use during vitrification. Among the tested exposure times to CPA solutions, a 2.5-fold increase proved effective in achieving adequate intracellular CPA concentration. The same treatment was also suitable to achieve post-warming maturation and cleavage rates similar to those obtained with standard 2D vitrification. Strategies aimed at improving cytoplasmic competence and embryo development are needed to fully exploit the advantages of 3D cryopreservation in the context of oocyte banking.

## Data Availability

The datasets presented in this study can be found in online repositories. The names of the repository/repositories and accession number(s) can be found below: the raw data supporting the conclusions of this article will be made available upon manuscript publication in the University of Milan Dataverse, at https://doi.org/10.13130/RD_UNIMI/KVKSBW.
