# Supplementary material for: Development of a three-dimensional vitrification protocol for domestic cat cumulus-oocyte complexes and comparison with standard vitrification
Source: Front Vet Sci. 2026 Apr 8;13:1807486. doi: 10.3389/fvets.2026.1807486 (PMC13099318; doi:10.3389/fvets.2026.1807486)
Supplement: Supplementary file 1 [file Data_Sheet_1.docx]

Supplementary Material

# Supplementary Figures

## Supplementary Figure 1

**
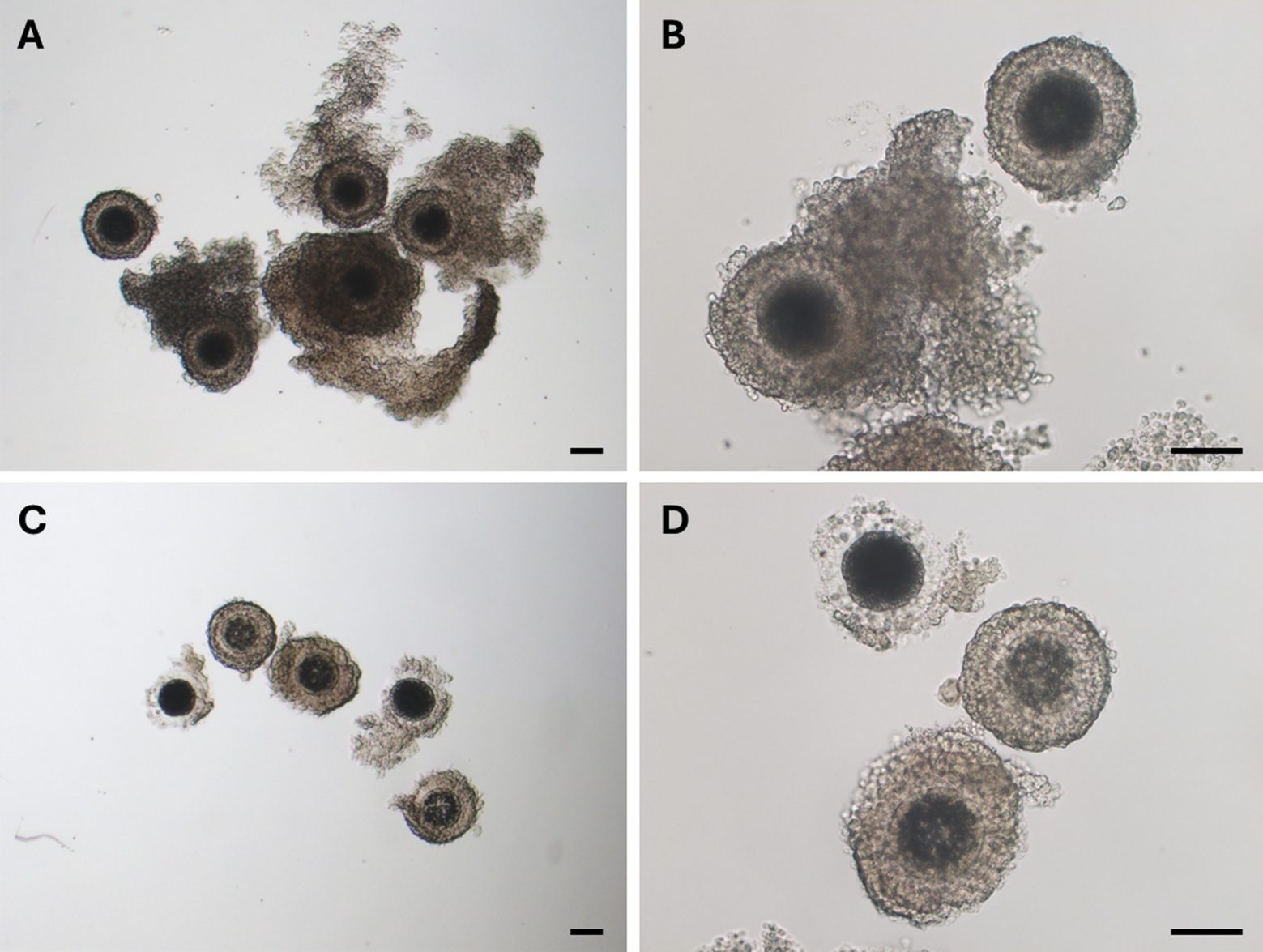
**

**Supplementary Figure 1.** Representative pictures of domestic cat cumulus-oocytes complexes (COCs) after collection. Micrographs at different magnifications of: (A, B) good quality, grade I COCs, selected for the experiments, and (C, D) Bad quality COCs, not included in the experiments. Scale bar: 100μm.

## Supplementary Figure 2

**
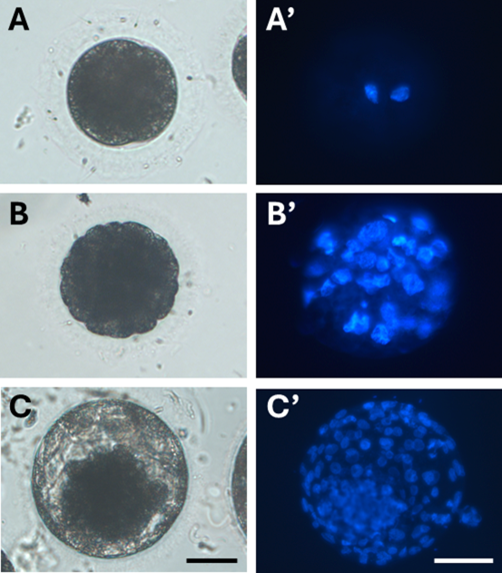
**

**Supplementary Figure 2.** Representative pictures of domestic cat embryos at different stages of development, with bright field (left column, X) and fluorescence visualization after Hoechst staining (right column, X’). (A, A’) Cleaved embryo at the 2-cells stage; (B, B’) Morula; (C, C’) Blastocyst. Scale bars: 50μm.
